# Correction: Characteristics of astigmatism before and 1 month after blepharoptosis surgery in patients with acquired ptosis

**DOI:** 10.1371/journal.pone.0336805

**Published:** 2025-11-13

**Authors:** Kazuhiko Dannoue, Seiji Takagi, Keiko Uemura, Anna Takei, Tomohiko Usui

In Table 1, the value in “Number of eyes” under “AP” column and the category name “MRD (mm)” and its values are incorrect. It should have been “84” and “MRD-1 (mm)” respectively. Please see the correct Table 1 here.

**Table 1:** Demographic data of the age-related ptosis (AP) and contact lens-related ptosis (CLP) groups.

	AP			CLP			P-value
Number of eyes	84			23			–
Age (years)	73.8 ± 7.6			47.7 ± 6.6			<0.01^*^
Sex (male:female)	13:32			3:10			>0.05^**^
	**Pre-operative value**	**Post-operative value**	**P-value** ^***^	**Pre-operative value**	**Post-operative value**	**P-value** ^***^	–
MRD-1 (mm)	0.78 ± 0.9	3.22 ± 1.2	<0.001	0.70 ± 0.5	3.85 ± 0.5	<0.001	–
Visual acuity (logMar)	0.05 ± 0.92	0.04 ± 0.89	>0.05	−0.08 ± 1.00	−0.08 + 1.15	>0.05	–

*Mann–Whitney U test,

**chi-square test, and

***student’s t-test

In Table 2, there are errors in the “Postoperative (cases/%)” group of contact lens-related ptosis (CLP). Please see the correct Table 2 here.

**Table 2:** Shift in the type of astigmatism postoperatively in the acquired ptosis (AP) and contact lens-related ptosis (CLP) groups.

AP		CLP	
Preoperative (cases)	Postoperative (cases/%)	Preoperative (cases)	Postoperative (cases/%)
WTR (21)	WTR (15/71.4)	WTR (20)	WTR (19/95.0)
	OA (5/23.8)		OA (0/0)
	ATR (1/4.8)		ATR (1/5.0)
OA (19)	OA (9/47.3)	OA (2)	OA (0/0)
	WTR (5/26.3)		WTR (2/100)
	ATR (5/26.3)		ATR (0)
ATR (44)	ATR (25/56.8)	ATR (1)	ATR (0)
	OA (14/31.8)		OA (1/100)
	WTR (5/11.3)		WTR (0)

The shaded cells represent a change in the type of astigmatism postoperatively. WTR: With-the-rule; ATR: Against the rule; OA: Oblique astigmatism.

Fig 1 is incorrect. Please see the correct Fig 1 here.

**Fig 1:**
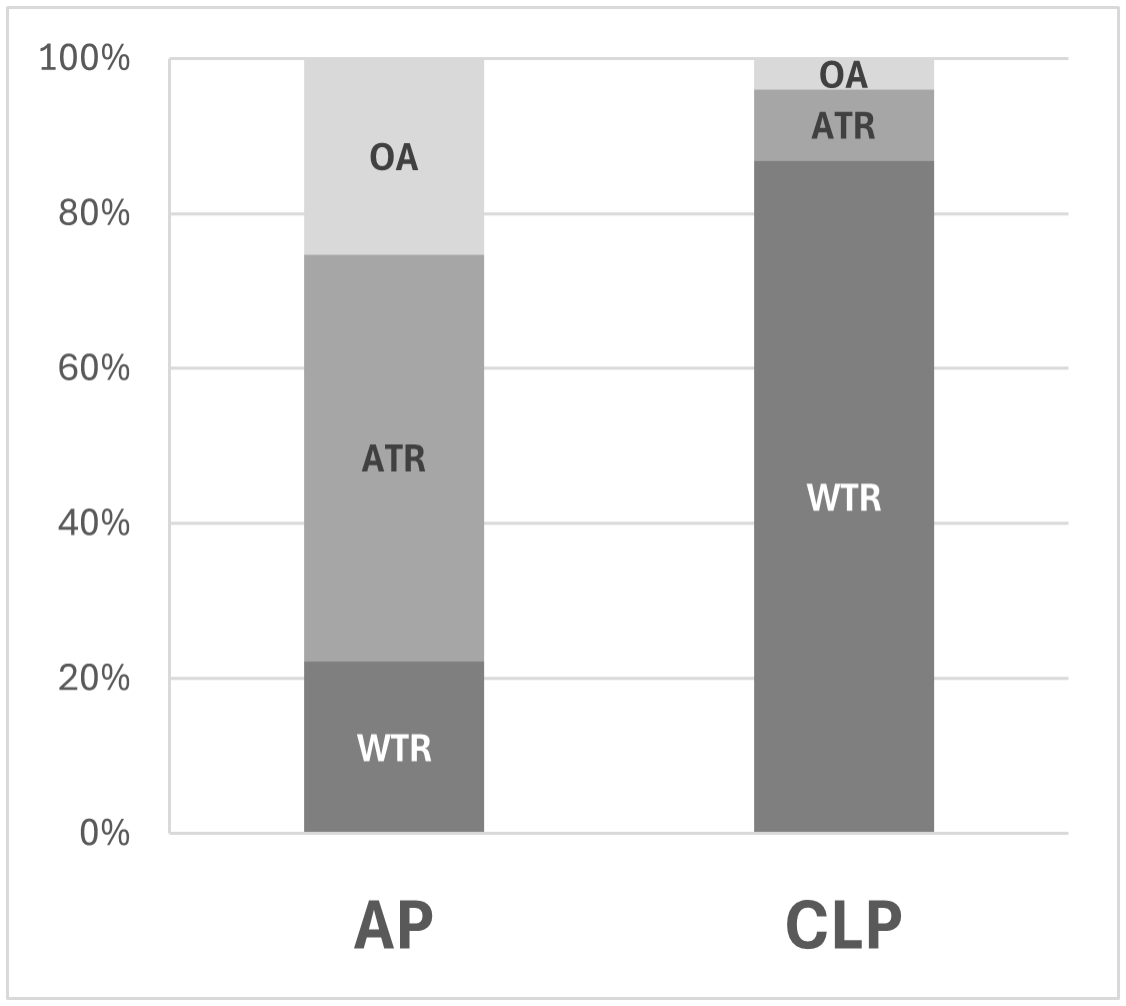
Proportions of the preoperative astigmatism type in the acquired ptosis (AP) and contact lens-related ptosis (CLP) groups. WTR: With-the-rule; ATR: Against the rule; OA: Oblique astigmatism.

In the Abstract section, there is an error in the seventh, tenth, eleventh and fifteenth sentences. The correct seventh sentence is: The correlation between SIA and margin reflex distance (MRD-1) was calculated. The correct tenth sentence is: The MRD-1 increased significantly after treatment in both groups. The correct eleventh sentence is: The proportions of WTR, ATR, and OA were 22%, 52%, and 25%, and 86%, 9%, and 4% in the AP and CLP groups, respectively. The correct fifteenth sentence is: There was no significant correlation between SIA calculated using the Cravy and Jaffe methods and MRD-1.

In the Patients subsection of Introduction, there is an error in the sixth sentence. The correct sentence is: Ptosis severity was evaluated using the margin reflex distance (MRD-1), a measurement of the distance from the middle upper eyelid to the corneal light reflex [29].

In the Statistical analyses subsection of Patients and Methods, there is an error in the fifth sentence. The correct sentence is: The association among the individual SIA was calculated using the Cravy method, the Jaffe method, and the preoperative MRD-1.

In the Results section, there is an error in the second and fifth sentences of the first paragraph. The correct second sentence is: One hundred and eight eyes from 58 patients (AP group: 84 eyes from 45 patients; females, 32; age, 73.8 ± 7.6 years; CLP group: 23 eyes from 13 patients; females, 10; age, 47.7 ± 6.6 years) were included. The correct fifth sentence is: The MRD-1 increased significantly after treatment in both the AP group (0.78 ± 0.9 mm to 3.22 ± 1.2 mm; P < 0.001) and CLP group (0.70 ± 0.5 mm to 3.85 ± 0.5 mm; P < 0.001).

In the Results section, there is an error in the second paragraph. The correct paragraph is: The proportions of WTR, ATR, and OA were 22%, 52%, and 25% and 86%, 9%, and 4% in the AP and CLP groups, respectively (Fig 1).

In the Results section, there is an error in the third and fifth sentences of the third paragraph. The correct third sentence is: WTR changed to OA in 23.8% and ATR in 4.8% of the patients, OA changed to WTR in 26.3% and ATR in 26.3% of the patients, and ATR changed to WTR in 11.3% and OA in 31.8% of the patients. The correct fifth sentence is: WTR changed to ATR in 5.0%, OA changed to WTR in 100%, and ATR changed to OA in 100% of the patients.

In the Discussion section, there is an error in the eighth sentence of the first paragraph. The correct eighth sentence is: WTR was most frequent in the CLP group, while the proportions of WTR and OA were 22% and 25%, respectively, in the AP group.

In the Discussion section, there is an error in the eighth sentence of the third paragraph. The correct eighth sentence is: We observed similar results in terms of the type of astigmatism: 41% of patients in the AP group and 13% of patients in the CLP group showed a change in the type of astigmatism postoperatively (Table 2).
