# Supplementary material for: Characteristics of astigmatism before and 1 month after blepharoptosis surgery in patients with acquired ptosis
Source: PLoS One. 2021 Oct 28;16(10):e0258688. doi: 10.1371/journal.pone.0258688 (PMC8553058; doi:10.1371/journal.pone.0258688)
Supplement: S1 Dataset — (PDF) [file pone.0258688.s002.pdf]

AP					Keratometric				
No	sex	R/L	age		Ks	Ks Axis	Kf	Kf Axis	MRD
1	F	R	63	Pre	44.73	86	44.07	176	2
2		L		Pre	44.49	86	43.95	176	2.5
3	F	R	82	Pre	44.07	139	43.71	49	1
4		L		Pre	43.99	172	43.40	82	1
5	M	L	68	Pre	45.88	174	45.12	84	0
6	M	R	87	Pre	45.56	146	44.48	56	0
7		L		Pre	45.96	11	43.37	101	1
8	F	R	71	Pre	47.46	98	46.53	8	2
9		L		Pre	47.15	96	46.60	6	2
10	M	L	79	Pre	46.95	62	45.55	152	1
11	F	R	85	Pre	47.27	7	45.35	97	0
12		L		Pre	45.89	174	45.61	84	0
13	F	R	76	Pre	44.18	163	43.68	73	1
14		L		Pre	45.02	35	43.92	125	1
15	M	R	71	Pre	45.61	96	45.10	6	0.5
16		L		Pre	45.08	83	44.24	173	0.5
17	F	R	81	Pre	46.74	155	43.80	65	3
18		L		Pre	43.91	145	43.76	55	2
19	F	R	71	Pre	43.98	91	42.81	1	0
20		L		Pre	43.21	121	42.12	31	0.5
21	F	R	81	Pre	46.73	23	42.97	113	1
22		L		Pre	43.64	8	43.15	98	0
23	F	R	77	Pre	45.98	121	44.81	31	0
24		L		Pre	45.91	27	45.26	117	0
25	F	R	87	Pre	45.70	8	44.48	98	0
26		L		Pre	46.18	14	44.37	104	0
27	F	R	70	Pre	45.67	38	45.37	128	0.5
28		L		Pre	45.50	17	45.15	107	0.5
29	F	R	80	Pre	45.68	163	44.60	73	
30		L		Pre	45.53	170	45.27	80	0.5
31	F	R	68	Pre	44.27	113	43.87	23	1
32		L		Pre	44.60	152	43.76	62	1
33	M	R	77	Pre	48.82	83	47.50	173	1
34		L		Pre	48.63	118	47.50	28	0.5
35	M	R	67	Pre	45.96	149	45.39	59	0.5
36		L		Pre	45.63	6	44.48	96	0
37	F	R	74	Pre	46.38	20	42.69	110	0
38		L		Pre	43.60	163	42.10	73	0
39	F	R	55	Pre	44.15	84	43.37	174	0
40		L		Pre	43.64	148	43.04	58	1
41	F	R	59	Pre	44.02	104	42.47	14	1
42		L		Pre	44.78	84	43.34	174	1
43	F	R	81	Pre	45.60	163	44.55	73	0
44		L		Pre	47.05	37	45.06	127	0
45	F	R	81	Pre	42.64	6	40.82	96	-1
46		L		Pre	44.04	11	41.04	101	-1.5
47	F	R	79	Pre	46.31	10	45.92	100	1
48		L		Pre	46.82	51	46.75	41	1

49	F	R	79	Pre	44.79	17	44.32	107	0.5
50		L		Pre	44.65	149	44.07	59	-1
51	M	R	73	Pre	44.14	91	43.71	1	0
52		L		Pre	44.57	105	44.22	15	0.5
53	F	R	74	Pre	42.84	149	42.72	59	4
54		L		Pre	43.13	162	42.31	72	3.5
55	F	R	80	Pre	43.29	163	42.37	73	1
56		L		Pre	43.71	10	43.17	100	0.5
57	F	R	78	Pre	46.01	10	43.99	100	1.5
58		L		Pre	47.03	174	45.17	84	0.5
59	F	R	75	Pre	43.18	163	42.65	73	1
60	F	R	60	Pre	45.11	93	44.09	86	1
61	F	R	60	Pre	45.48	80	44.12	170	3
62		L		Pre	45.05	93	44.25	3	1
63	F	R	70	Pre	44.08	172	42.98	82	1
64		L		Pre	43.74	156	42.98	66	1
65	M	R	69	Pre	45.69	6	44.94	96	1
66		L		Pre	45.93	166	44.91	76	2
67	F	R	82	Pre	46.21	131	45.17	41	1
68		L		Pre	48.87	158	46.35	68	1
69	M	R	64	Pre	39.68	10	39.14	100	0
70		L		Pre	43.16	51	40.32	141	0
71	M	R	64	Pre	43.87	10	42.11	100	1
72		L		Pre	44.15	167	42.36	77	0
73	F	R	74	Pre	44.97	30	43.65	120	1
74		L		Pre	44.55	160	43.58	70	1
75	M	R	76	Pre	43.95	28	43.39	118	1
76	M	R	67	Pre	45.01	121	44.05	31	1
77		L		Pre	45.18	21	44.90	111	2
78	F	R	71	Pre	46.24	68	45.65	158	0
79		L		Pre	46.16	166	46.01	76	1.5
80	F	R	78	Pre	44.22	80	43.10	170	0
81		L		Pre	44.79	52	43.33	142	0
82	M	R	77	Pre	45.54	145	45.47	55	1
83		L		Pre	46.46	163	46.02	73	1.5
84	F	R	78	Pre	43.44	131	42.86	41	0.5
85		L		Pre	45.07	79	44.13	169	0

CLP					Keratometric				
No	sex	R/L	age		Ks	Ks Axis	Kf	Kf Axis	MRD
1	F	R	54	Pre	43.02	86	42.14	176	
2		L		Pre	42.36	131	41.85	41	0.5
3	F	R	53	Pre	45.09	107	43.93	17	0.5
4	M	R	33	Pre	45.01	89	41.18	179	0
5		L		Pre	43.66	96	41.11	6	1
6	F	R	53	Pre	47.72	96	45.46	6	1
7		L		Pre	47.49	87	44.60	177	0.5

8	F	R	50	Pre	43.00	79	41.51	169	0.5
9		L		Pre	42.43	113	41.73	23	1
10	F	R	48	Pre	45.29	86	42.61	176	1
11		L		Pre	44.76	93	42.39	3	1
12	F	R	44	Pre	45.08	86	43.52	176	1
13	F	R	50	Pre	46.62	98	45.69	8	1.5
14		L		Pre	51.80	35	46.89	125	1.5
15	F	R	57	Pre	43.53	108	41.56	18	1
16		L		Pre	43.59	82	41.22	172	1
17	F	R	40	Pre	42.84	107	41.41	17	0
18		L		Pre	43.44	86	41.24	176	0
19	F	R	44	Pre	45.77	69	44.73	159	1.5
20		L		Pre	46.32	94	44.72	4	0
21	F	R	53	Pre	44.68	96	43.77	6	0
22		L		Pre	44.91	93	44.27	3	0
23	M	R	41	Pre	41.99	10	41.74	100	1

	Keratometric						jafee	
	Ks	Ks Axis	Kf	Kf Axis	Ave	MRD	magnitude	axis
1M	44.23	51	43.81	141	44.02	6	0.657884	14.43172
1M	44.22	53	44.10	143	44.16	6	0.503276	2.290619
1M	44.47	37	43.72	127	44.09	4	1.078859	40.90019
1M	44.41	131	43.92	41	44.16	4	0.725601	103.5149
1M	46.24	152	45.58	62	45.91	3	0.54531	112.6102
1M	45.29	160	43.99	70	44.64	1	0.615256	7.366212
1M	45.99	11	42.80	101	44.39	3	0.61	11
1M	48.02	104	46.51	14	47.26	3.5	0.630694	112.9266
1M	47.87	96	46.79	6	47.33	3.5	0.498	96
1M	46.18	48	45.01	138	45.59	2.5	0.734052	176.2211
1M	47.65	10	45.70	100	46.67	2.5	0.204744	49.29303
1M	45.76	30	45.43	120	45.59	2.5	0.360824	53.78161
1M	44.07	96	43.62	6	43.84	4	0.865416	83.9826
1M	45.04	72	44.02	162	44.53	4	1.277447	99.93341
1M	45.68	93	45.12	3	45.40	3.5	0.075027	70.36077
1M	45.05	90	44.35	180	44.70	3	0.233521	149.758
1M	44.49	121	44.05	31	44.27	2.5	2.804999	69.1814
1M	44.35	25	43.50	115	43.92	2	0.940266	29.23718
1M	43.34	86	42.74	176	43.04	2	0.588413	6.099502
1M	43.55	124	42.28	34	42.91	1	0.218098	139.747
1M	43.76	7	43.25	97	43.51	3	3.338453	115.3217
1M	43.51	136	43.07	46	43.29	3	0.741651	115.5725
1M	46.27	113	45.55	23	45.91	5	0.526711	42.06747
1M	46.26	132	45.08	42	45.67	5	1.782392	126.6652
1M	45.71	38	44.36	128	45.03	3	1.289922	65.49645
1M	46.18	6	44.49	96	45.33	3.5	0.501391	138.1451
1M	45.51	44	45.21	134	45.36	4	0.064533	81.56741
1M	45.49	10	45.12	100	45.30	2	0.089517	151.683
1M	45.16	17	44.91	7	45.04	3	1.013219	66.38761
1M	46.36	62	45.35	152	45.86	2	1.238644	65.68065
1M	44.77	127	43.88	37	44.33	3	0.561983	137.0149
1M	44.65	163	43.56	73	44.10	3	0.43878	6.262853
1M	51.19	87	47.48	177	49.33	2	2.400092	89.21158
1M	47.98	145	47.76	55	47.87	2.5	1.016392	22.95738
1M	46.07	107	45.84	17	45.95	2	0.59194	70.3661
1M	45.99	42	44.47	132	45.23	0.5	1.593446	63.43803
1M	46.67	1	42.51	91	44.59	2.5	2.594058	150.4321
1M	43.60	174	42.26	84	42.93	2.5	0.556024	41.17801
1M	44.66	80	43.35	170	44.01	4	0.557883	74.46263
1M	44.33	120	43.77	30	44.05	4	0.474349	100.505
1M	44.39	107	42.54	17	43.47	3	0.356811	120.4086
1M	44.73	86	43.26	173	44.00	3	0.105891	121.7762
1M	46.53	120	45.24	30	45.88	3.5	1.603967	99.37858
1M	47.42	35	45.64	125	46.53	3.5	0.248075	142.1103
1M	42.64	169	40.98	79	41.81	2	1.028894	128.2239
1M	43.73	169	41.09	73	42.41	2	2.133855	130.6263
1M	45.56	82	44.97	172	45.27	3.5	0.934082	89.10313
1M	44.59	11	43.78	101	44.18	3.5	0.800817	8.530855

1M	45.11	23	44.61	13	44.86	3	0.105691	56.80151
1M	45.04	153	44.55	63	44.79	2	0.116755	41.13074
1M	44.64	105	43.92	15	44.28	1	0.413028	119.6297
1M	44.97	30	44.58	120	44.78	1	0.71486	22.9149
1M	42.82	172	42.44	82	42.63	4.5	0.323417	179.0809
1M	43.15	155	42.53	65	42.84	4.5	0.264959	89.23913
1M	43.37	138	42.90	48	43.14	3	0.723792	87.91528
1M	43.71	163	43.14	73	43.42	3	0.508782	132.5036
1M	46.20	7	44.35	97	45.27	4	0.264279	123.5153
1M	47.92	37	45.78	127	46.85	3	2.735669	58.35355
1M	43.41	148	42.89	58	43.15	4.5	0.271932	109.4822
1M	45.12	92	44.12	94	44.64	3	0.78552	88.5678
1M	45.95	89	44.12	179	45.03	4	0.676027	108.3865
1M	45.24	86	44.06	176	44.65	4	0.45679	73.4661
1M	44.04	6	42.80	96	43.42	3.5	0.582167	37.25318
1M	43.27	172	42.60	82	42.94	3.5	0.398888	34.55777
1M	46.47	125	45.63	35	46.05	4	1.382443	111.3038
1M	45.81	38	45.45	128	45.63	4.5	1.160891	67.24431
1M	45.98	142	45.22	52	45.60	2.5	0.439895	20.83451
1M	45.46	14	44.46	104	44.96	2	2.406856	56.3625
1M	39.15	38	39.00	128	39.08	4	0.460066	91.62132
1M	43.31	55	40.56	145	41.94	4	0.398705	103.7732
1M	44.07	10	42.96	100	43.52	4.5	0.65	100
1M	44.03	163	42.52	73	43.27	4.5	0.361951	94.74661
1M	45.04	28	43.87	118	44.46	6	0.164889	134.9735
1M	44.72	170	44.28	80	44.50	6	0.576523	62.43445
1M	44.05	49	43.53	139	43.79	4	0.388835	86.25556
1M	44.88	121	43.86	31	44.37	4.5	0.06	121
1M	44.74	169	44.34	79	44.54	5	0.367096	147.3606
1M	46.48	82	45.69	172	46.08	3	0.389059	104.2086
1M	46.89	107	46.17	17	46.53	3	0.80144	102.244
1M	44.26	83	43.16	173	43.71	2	0.11789	131.377
1M	45.18	94	43.05	4	44.11	2	2.458297	112.2456
1M	46.06	44	45.18	134	45.62	3.5	0.955263	44.7865
1M	46.27	146	45.79	56	46.03	2	0.270321	114.5941
1M	43.63	128	42.75	38	43.19	3.5	0.30918	122.3458
1M	45.18	82	44.55	172	44.86	3.5	0.310791	162.7849

	Keratometric						Jafee	
	Ks	Ks Axis	Kf	Kf Axis	Ave	MRD	magnitude	axis
1M	43.04	79	42.33	169	42.68	3.5	0.256941	16.97567
1M	42.45	107	41.81	17	42.13	4	0.476463	80.65095
1M	44.61	84	43.99	174	44.30	3.5	0.852753	33.05129
1M	43.93	91	41.29	1	42.61	3	1.220405	174.6605
1M	43.49	91	41.13	1	42.31	3	0.467925	36.57001
1M	47.53	79	45.45	169	46.49	4	1.280516	38.63728
1M	47.58	83	45.46	173	46.52	4	0.843891	7.232254

1M	43.71	83	41.90	173	42.80	4	0.393563	98.89807
1M	43.11	96	41.93	6	42.52	3.5	0.720619	79.28381
1M	45.35	90	42.43	180	43.89	4.5	0.458166	117.2482
1M	45.24	100	42.54	10	43.89	4.5	0.702959	127.1552
1M	44.45	93	43.19	3	43.82	3.5	0.454723	154.9532
1M	46.25	107	45.58	17	45.92	3	0.358599	170.3674
1M	47.38	69	45.82	159	46.60	4	4.560432	115.6928
1M	43.29	100	41.64	10	42.47	4	0.595179	42.9154
1M	43.33	77	41.16	167	42.24	4	0.448357	20.59326
1M	43.67	105	41.44	15	42.56	4.5	0.819468	101.5287
1M	44.11	91	41.32	1	42.72	4.5	0.722656	106.9569
1M	45.96	66	45.15	156	45.55	3.5	0.249258	168.9287
1M	45.61	93	44.61	3	45.11	3	0.591639	5.690857
1M	44.79	172	44.68	82	44.74	4.5	1.008447	4.53232
1M	45.31	107	44.29	17	44.80	4.5	0.553554	123.4368
1M	41.97	44	41.71	134	41.84	3.5	0.285309	71.16745

c r a v y
-0.124389
-0.408299
1.0718476
0.5599823
0.4124008
-0.48824
0.7151861
0.3083262
0.4432167
-0.403315
0.1193113
0.6999865
0.7258242
-0.246726
0.0760278
-0.236109
1.5725841
1.1688792
-0.50901
-0.030218
-4.361895
-0.57558
-0.022275
-0.800361
0.5170393
-0.336719
0.0172378
-0.020895
1.0292168
1.5849564
-0.042037
-0.372232
2.4169094
-0.521255
0.3477183
0.8937412
-0.497552
-0.20107
0.6713085
0.3231609
0.1065804
-0.013671
1.1722816
-0.307917
-3.31303
-5.593344
0.2145725
0.8512207

0.0687401
-0.015703
0.100829
0.2852625
-0.294293
0.2265849
0.5826711
-1.015593
-0.278412
4.6523519
0.1864575
0.116764
0.2745746
0.5130766
2.2990269
-0.190127
-0.62046
1.2482633
-0.233582
2.6047123
-0.389394
-0.150434
-0.752996
0.3389211
-0.207299
0.2228321
-0.023755
0.0205278
-0.670165
0.1372201
0.5872879
-0.071614
-0.087168
1.2756487
0.1559658
0.09445
-0.379248

Cravy
-0.106812
0.367966
-0.077761
-1.312909
0.0489696
0.4272821
-0.674729

0.2701621
0.6829528
0.059581
-0.043122
-0.47269
-0.346683
-4.809938
0.0735954
-0.085543
0.6340632
0.3829537
-0.274198
-0.52892
-0.903514
0.0782232
0.0780255
